# Data on characterization of crude bio-oils, gaseous products, and process water produced from hydrothermal liquefaction of eight different algae

**DOI:** 10.1016/j.dib.2018.05.144

**Published:** 2018-06-02

**Authors:** Shi-Kun Yang, Yu-Ping Xu, Pei-Gao Duan

**Affiliations:** Henan Polytechnic University, China

## Abstract

The characterization of products produced from hydrothermal liquefaction of algal biomass is helpful to better understand the effect of different kinds of raw materials on the properties of the product fractions. The data presented in this article are related to the research article entitled “Integration of hydrothermal liquefaction and supercritical water gasification for the improvement of energy recovery from algal biomass” (Duan et al., 2018) [1]. In this data article, the compositions of gaseous products produced from hydrothermal liquefaction of eight different algae feedstocks at 350 °C for 60 min were analyzed by gas chromatography. The molecular and elemental compositions of the crude bio-oils produced from hydrothermal liquefaction of eight different algae feedstocks at 350 °C for 60 min were analyzed by comprehensive two-dimensional gas chromatography*-*time-of-flight mass spectrometry and organic elemental analyzer. The color of aqueous phases before and after they were subjected to supercritical water gasification was recorded by a high-resolution camera.

**Specifications Table**

**Table:** 

Subject area	*Energy recovery*
More specific subject area	*Hydrothermal liquefaction of algal biomass and supercritical water gasification of the HTL process water*
Type of data	*Tables and figures*
How data was acquired	*Gas Chromatography*; *Comprehensive two-dimensional gas chromatography*–*time-of-flight mass* spectrometry*; Organic elemental analyzer; high resolution camera.*
Data format	*Raw, filtered, analyzed*
Experimental factors	*The gaseous products produced from the hydrothermal liquefaction of algal were collected by using a 0.5 L gas aluminum-plastic composite film bag, then waiting for analysis; The bio-oil samples were prepared by dissolved the bio-oils in dichloromethane at a concentration of 20 (wt./vol) %.*
Experimental features	*The gaseous products were analyzed by using a gas chromatograph equipped with a thermal conductivity detector (TCD). The molecular compositions of the bio-oils were determined by comprehensive two-dimensional gas chromatography*–*time-of-flight mass spectrometry; the C, H,O,N, and S contents of the bio-oils were analyzed by an organic elemental analyzer.*
Data source location	*College of Chemistry and Chemical Engineering, Department of Energy and Chemical Engineering, Henan Polytechnic University, No. 2001, Century Avenue, Jiaozuo, Henan 454003, P.R. China*
Data accessibility	*Data are available within this article*

**Value of the data**

• The data can give the readers a deeper understanding of the ingredients of bio-oils produced from the HTL of different algae and also help to infer the possible molecular composition of the organics in the HTL process water because this was the solution saturated with compounds existing in the bio-oil.
• The data can assist researchers to discern the effect of biochemical compositions of algal biomass feedstocks on the elemental compositions of their resulting bio-oils.
• The data can help the researchers to understand the effectiveness of supercritical water gasification on processing the HTL process water more intuitively.

## 1 Data

**Fig. 1:**
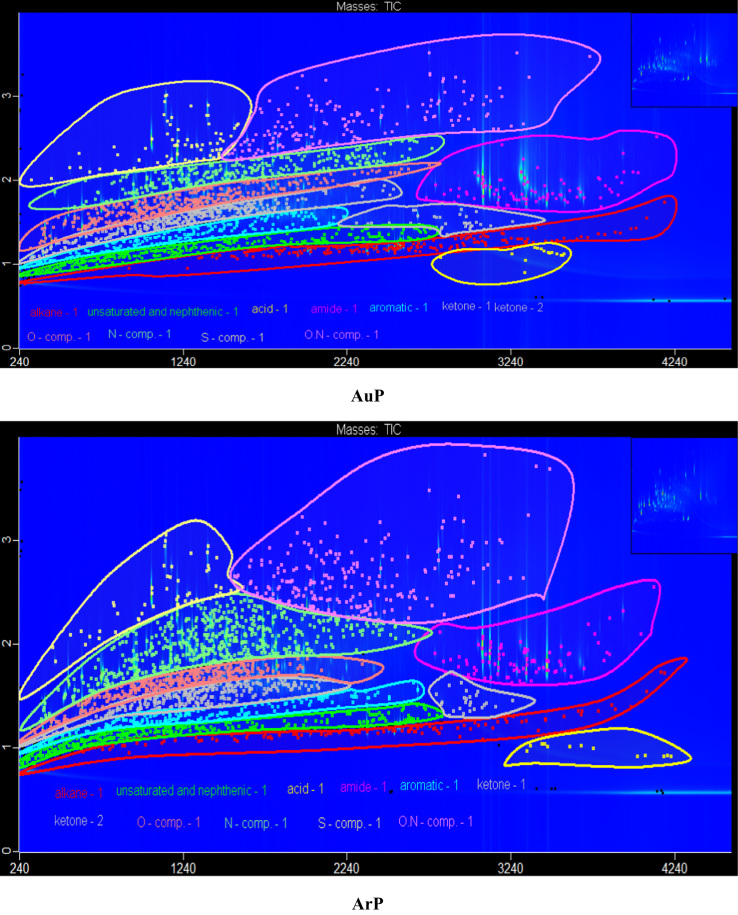

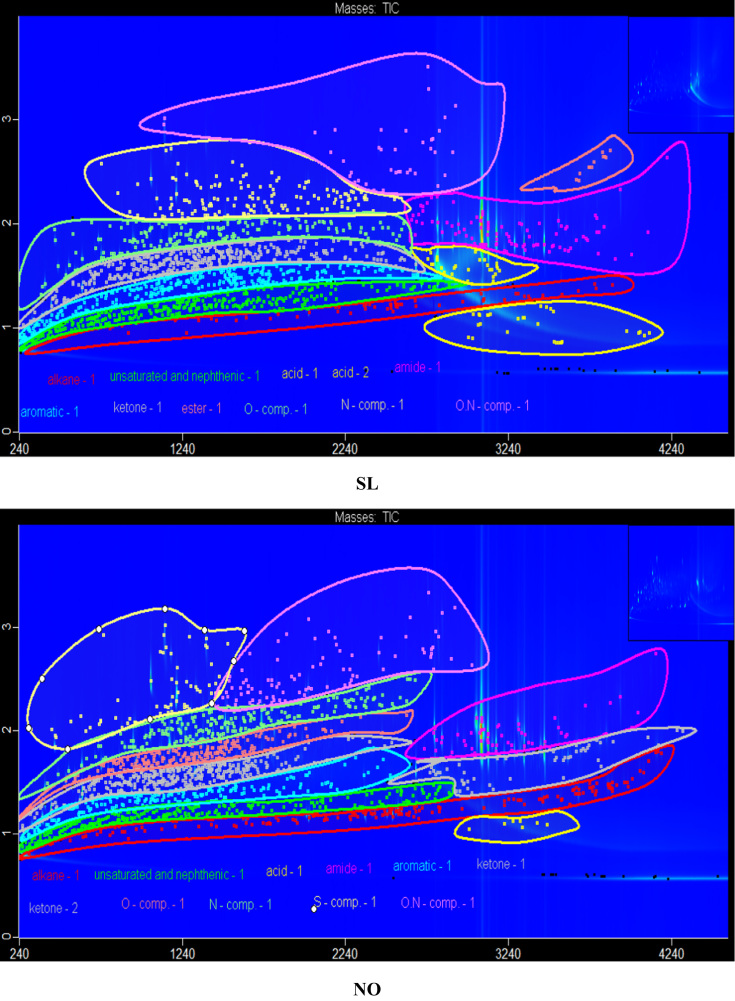

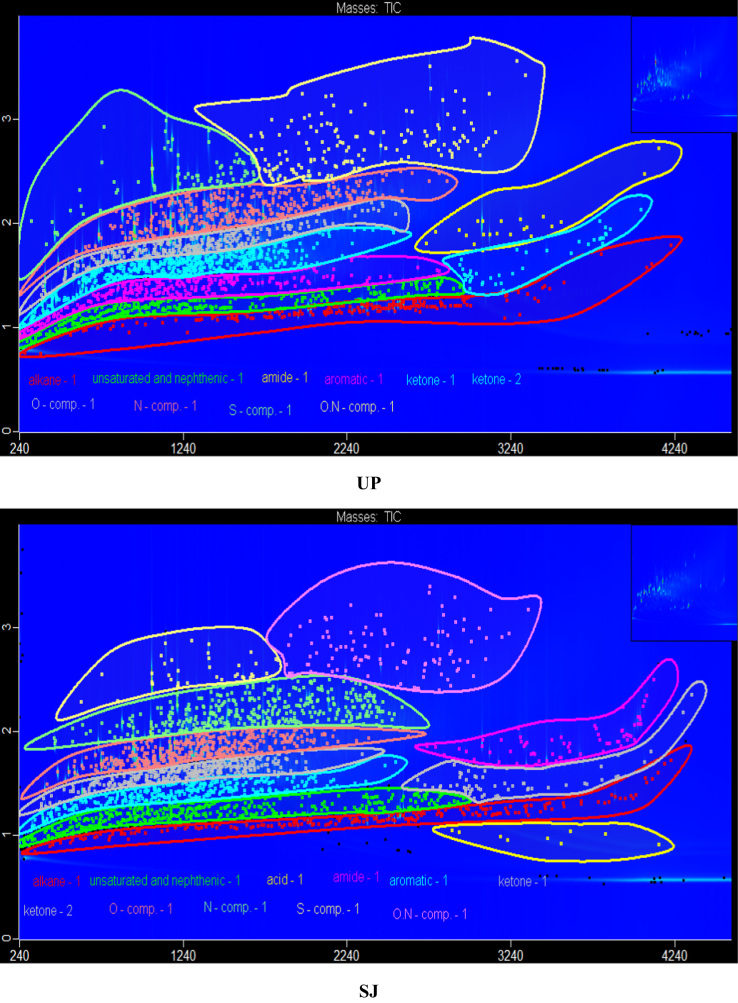

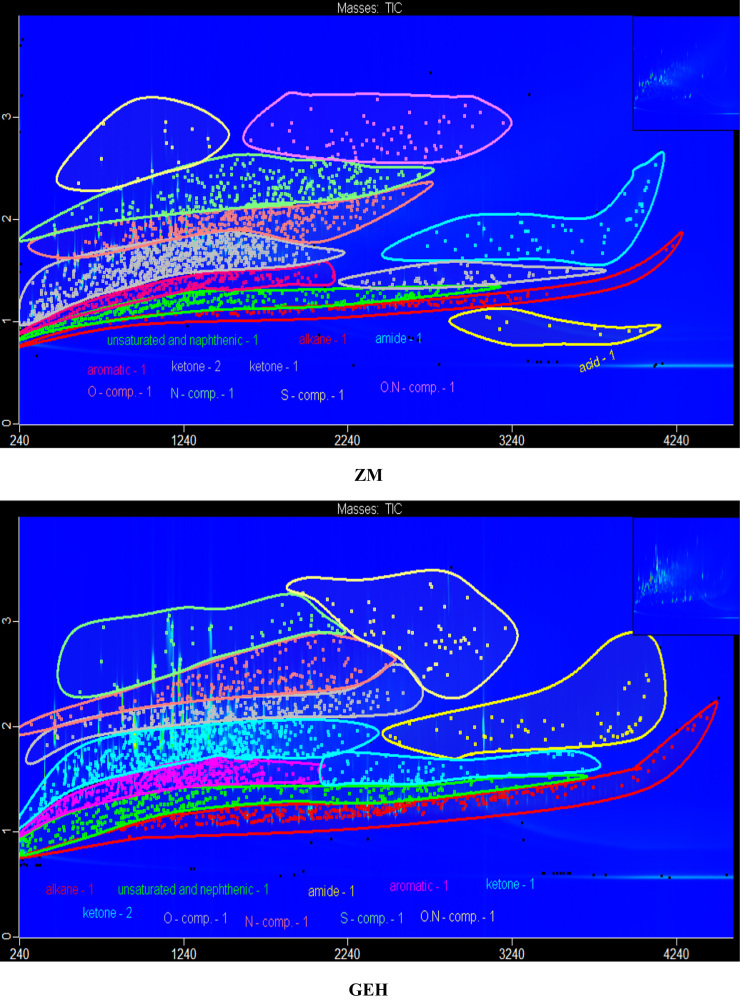
GC×GC-TOF-MS chromatograms of the crude bio-oils produced from the HTL of different algal biomass feedstocks.

**Fig. 2:**
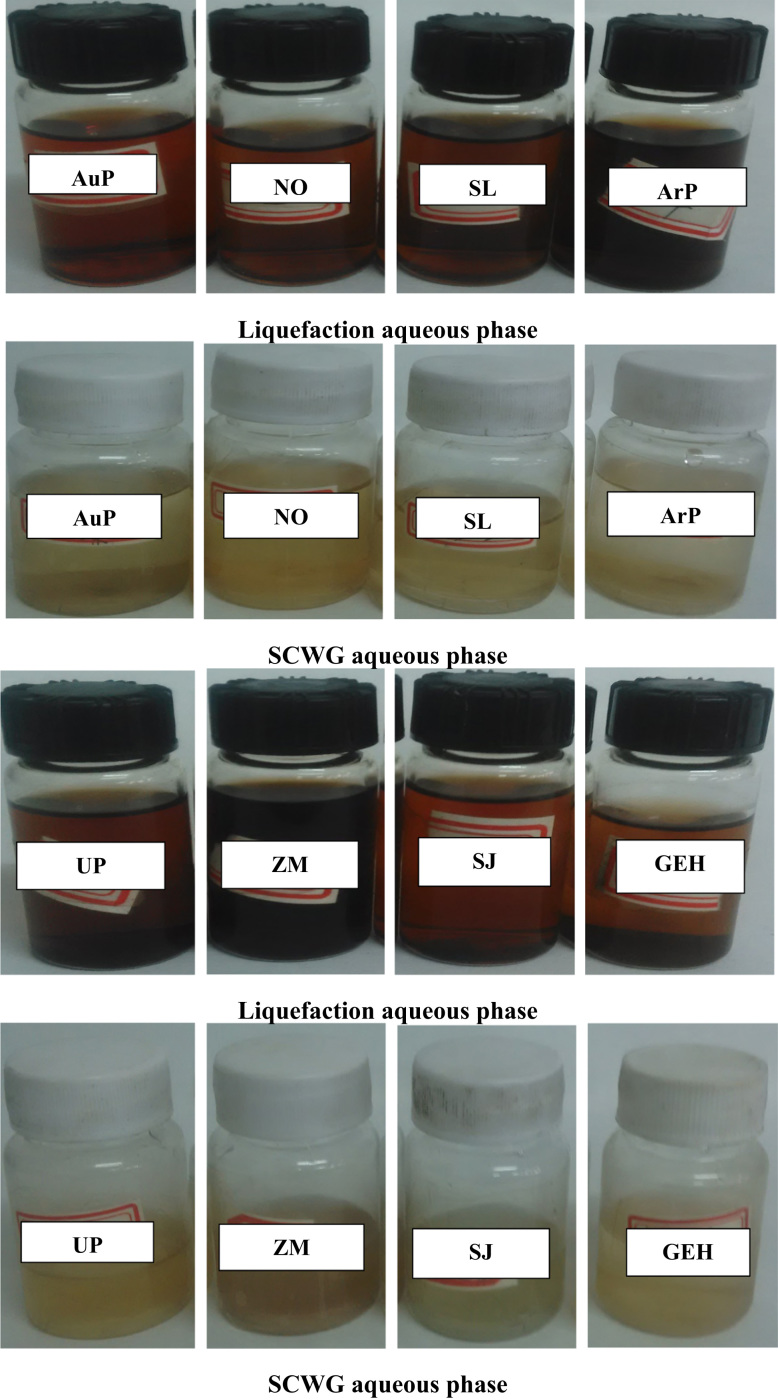
Comparison of the color of aqueous phases produced from HTL and SCWG process.

The data in Fig. 1 presents the chromatograms of the crude bio-oils produced from hydrothermal liquefaction of four microalgae (*Nannochloropsis oceanica*, *Auxenochlorella pyrenoidosa*, *Arthrospira platensis*, and *Schizochytrium limacinum)* and four macroalgae (*Ulva prolifera*, *Saccharina japonica* (*Areschoug*), *Zostera marina*, and *Gracilaria eucheumoides harvey*) at 350 °C for 1 h [1]. The crude bio-oil mainly consisted of long chain hydrocarbons, aromatics, saturated and unsaturated hydro-carbons, alcohols, esters, ketones, amides, S–, O–, N–, and N,O-containing compounds. Fig. 2 shows a comparison of the color of the HTL process waters before and after the supercritical water gasification treatment process. The data in Table 1 provides the elemental compositions and other properties of the crude bio-oils produced from the HTL of eight different algal biomass feedstocks at 350 °C for 1 h.

**Table 1:** Ultimate analyses (wt%) and other properties of the bio-oils produced from HTL of different algae.

	N	C	H	O	S	H/C	O/C	HHV (MJ/Kg)
AuP	6.11	75.26	9.27	4.92	0.87	1.48	0.05	37.88
ArP	7.16	75.67	9.40	4.45	0.86	1.49	0.04	38.28
SL	2.44	76.33	10.23	4.45	0.49	1.61	0.04	39.67
NO	4.91	73.94	9.33	4.86	0.72	1.51	0.05	37.52
UP	5.25	74.18	8.21	5.18	1.30	1.33	0.05	36.00
SJ	4.98	75.65	8.64	6.32	1.95	1.37	0.06	36.97
ZM	3.54	75.98	8.61	6.92	0.72	1.36	0.07	36.81
GEH	4.66	72.66	8.81	7.43	4.78	1.46	0.08	36.27

## 2 Experimental design, materials and methods

### 2.1 Gas chromatography analysis

The gaseous products were analyzed using a GC-7900 gas chromatograph (Shanghai Techcomp Scientific Instrument Co., Ltd., Shanghai, China) equipped with a thermal conductivity detector (TCD). The gas component was separated using a 15 ft×1/8 in i.d. stainless-steel column packed with 60×80 mesh Carboxen 1000 (Supelco). Argon (15 mL/min) served as the carrier gas for the analysis. The temperature of the column was maintained at 70 °C for 120 min. The reactor gas-collection bag was connected to the GC gas sampling valve, and the gases in the reactor flowed into the sample loop as the reactor valve was opened slowly (and slightly) to allow a predetermined amount of sample to exit. The gas sample was then sent to the column via a switching valve. After the switching valve was closed, the reactor valve was also closed. To ensure that the GC sample was representative of the gas mixture, we conducted a subsequent analysis. Thus, two consecutive analyses of the gas mixture were carried out for each run, and the values were presented as the average values of these two runs. Gas standards were purchased from Changzhou Jinghua Industrial Gases Co., Ltd. (Changhong Rd, Wujin, Changzhou, Jiangsu, China) and were analyzed to generate a calibration curve for each component, which in turn was used to calculate the mole fraction of each component in the reactor samples. The amount of helium added to the reactor was then used as an internal standard to determine the molar amount of each constituent.

### 2.2 Comprehensive two-dimensional gas chromatography–time-of-flight mass spectrometry analysis

The molecular composition of the bio-oil and organic matter in the WSPs were analyzed by comprehensive *two-dimensional gas chromatography-time-of-flight mass spectrometry (GC×GC-TOF-MS)* with a LECO Pegasus-IV CTDMGC/TOF-MS system equipped with 2 GC columns: a non-polar Rxi-5Sil MS (30 m×0.25 mm ID×0.25 μm film thickness) and a polar Rxi-17 (1 m×0.1 mm ID×0.1 μm film thickness). The samples were prepared by redissolution in ethanol at a concentration of 10 (wt/vol)%. Because some inorganic salts are insoluble in the ethanol, only the supernatant was collected and analyzed. The sample injection volume was 2 µL, and the column was initially held at 45 °C for 8 min. The temperature was ramped to 300 °C at 8 °C/min and held isothermally for 4–10 min. The m/z values ranged from 35 to 500, and identification of compounds was realized using Chroma TOF v4.51.6.0 by comparing the acquired spectra with the NIST11 database. Helium flowing at 2 mL/min served as the carrier gas.

### 2.3 Elemental analysis

An organic element analyzer (Flash 2000) (Thermo Fisher Scientific, Waltham, MA, U.S.A) was used to quantify C, H, O, N, and S in the sample. Dulong formula was employed to estimate the higher-heating value (HHV) of the algae and bio-oils as follows:

HHV=0.338×C+1.429(H−O/8)+0.095S where C, H, S, and O represent the weight percentage of carbon, hydrogen, sulfur, and oxygen in a sample, respectively.

## References

[1] Duan P.-G., Yang S.-K., Xu* Y.-P., Wang F., Zhao D., Weng Y.-J., Shi* X.-L. (2018). Integration of hydrothermal liquefaction and supercritical water gasification for the improvement of energy recovery from algal biomass. Energy.

