# Supplementary material for: Data on characterization of crude bio-oils, gaseous products, and process water produced from hydrothermal liquefaction of eight different algae
Source: Data Brief. 2018 Jun 2;19:1257–65. doi: 10.1016/j.dib.2018.05.144 (PMC6140826; doi:10.1016/j.dib.2018.05.144)
Supplement: Supplementary file 1 — Supplementary material [file mmc1.pdf]

Declaration of interest

We wish to confirm that there are no known conflicts of interest associated with this publication and there has been no significant financial support for this work that could have influenced its outcome.

Signed by all authors as follows:

Shi-kun Yang, Yu-Ping Xu, Peigao Duan

May. 27. 2018
